# A descriptive study of vancomycin use at Red Cross War Memorial Children’s Hospital, Cape Town

**DOI:** 10.4102/sajid.v38i1.528

**Published:** 2023-11-06

**Authors:** Leonore Greybe, Brian S. Eley, Hafsah D. Tootla, Anna M.M. Botha, Wisdom Basera, James J.C. Nuttall

**Affiliations:** 1 Department of Paediatrics and Child Health, Faculty of Health Sciences, University of Cape Town, Cape Town, South Africa; 2 Department of Medical Microbiology, Faculty of Pathology, National Health Laboratory Service (Red Cross War Memorial Children’s Hospital), Cape Town, South Africa; 3 Department of Pharmacy, Faculty of Health, Red Cross War Memorial Children’s Hospital, Cape Town, South Africa; 4 School of Public Health and Family Medicine, Faculty of Health Sciences, University of Cape Town, Cape Town, South Africa; 5 Burden of Disease Research Unit, South African Medical Research Council, Cape Town, South Africa

**Keywords:** vancomycin, paediatrics, AMS, antimicrobial stewardship, audit, TDM, therapeutic drug monitoring

## Abstract

**Background:**

Antimicrobial stewardship principles guide the clinical use of antimicrobials, including vancomycin, but paediatric vancomycin prescribing practices have not been evaluated in South Africa.

**Objectives:**

To document the use, prescribing practices and monitoring of intravenous vancomycin and the spectrum of bacteria isolated on microbiological culture in children treated with intravenous vancomycin during a 12-month period at Red Cross War Memorial Children’s Hospital (RCWMCH).

**Method:**

A retrospective audit of intravenous vancomycin use in children admitted to RCWMCH during 2019 was performed.

**Results:**

All 158 vancomycin prescription episodes for 143 children were included. Overall usage of intravenous vancomycin was 63 days of therapy per 1000 patient days (interquartile range [IQR]: 38–72). The median starting dose was 15 mg/kg per dose (IQR: 14–15) and median daily dose was 45 mg/kg per day (IQR: 43–60). Vancomycin was prescribed as empiric (127/158, 80%) and directed (31/158, 20%) treatment. The median duration of treatment for the directed group (7 days) was longer than the empiric group (4 days) (*p* = 0.001). Vancomycin serum trough concentrations were performed in 65/98 (66%) episodes where vancomycin treatment exceeded 3 days, with only 16/65 (25%) of these samples obtained before the fourth dose. Prolonged antibiotic treatment of 14 days or more was not associated with Gram-positive bacteria on culture (odds ratio [OR]: 1.02, 95% confidence interval [CI]: 0.17–4.2).

**Conclusion:**

Dosing errors, prolonged empiric treatment and inappropriate vancomycin monitoring were problems associated with vancomycin prescriptions.

**Contribution:**

The study identified multiple opportunities for improved vancomycin prescribing and monitoring. Further research and implementation of improved prescribing practices could contribute to the preservation of vancomycin as an effective antibiotic.

## Introduction

Children in low- and middle-income countries (LMICs) are leading consumers of antibiotics globally,^1^ and little progress has been made in mitigating the risk of antimicrobial resistance by improving stewardship in this setting.^2^ The World Health Organization (WHO) has classified 180 antibiotics used in human health into Access, Watch and Reserve categories. This classification is based on spectrum of activity, frequency of use and resistance potential. Vancomycin is a Watch antibiotic and should be prioritised for stewardship and surveillance for resistance.^3^ Vancomycin’s mechanism of action against Gram-positive bacteria is by inhibition of bacterial cell wall synthesis. It remains the treatment of choice for infection caused by β-lactam-resistant Gram-positive bacteria such as *Enterococcus faecium*, β-lactam-resistant *Streptococcus pneumoniae* and methicillin-resistant *Staphylococcus aureus* (MRSA).^4,5^

A pharmacist-led, multicentre, antimicrobial stewardship (AMS) initiative, across 47 private hospitals in South Africa, showed that 1 in every 15 antibiotic prescriptions required intervention when dosing errors and excessive duration were common.^6^ An audit on the impact of stewardship on vancomycin use in children indicated that non-approved indications for prescription, dosing errors and prolonged duration of treatment were problems.^7^ Real-time intervention and feedback on prescriptions of vancomycin have reduced overall vancomycin use and improved the safety and quality of care in tertiary hospitals.^8,9^

Achievement of adequate plasma and tissue vancomycin concentrations is essential for an optimal clinical response. The effectiveness of vancomycin to treat invasive infections due to Gram-positive bacteria is associated with the area under the concentration-time curve from 0 h to 24 h divided by the minimum inhibitory concentration (AUC_24_/MIC ratio) of the organism being targeted. For MRSA with a vancomycin MIC of 1 mg/L, an AUC_24_/MIC ratio > 400 is recommended.^10^ The 2011 Clinical Practice Guidelines produced by the Infectious Diseases Society of America (IDSA) recommended targeting vancomycin trough concentrations of 15 mg/L – 20 mg/L, which correlates with an AUC_24_/MIC ratio > 400, in adults with severe bacterial infections. The effectiveness and safety of targeting trough concentrations of 15 mg/L – 20 mg/L in children were not established at the time and a dose of 15 mg/kg per dose administered intravenously six-hourly was recommended.^11^ Pharmacokinetic modelling suggests that a trough concentration of 7 mg/L – 10 mg/L may be sufficient to achieve an AUC_24_/MIC of > 400 in over 90% of children with MRSA infection.^12^ Although optimal dosing and the role of therapeutic drug monitoring (TDM) in paediatric practice remain uncertain,^11,13^ current IDSA guidelines recommend individualised targeting of an AUC_24_/MIC ratio of 400–600 (assuming a vancomycin MIC of 1 mg/L) to achieve clinical efficacy while improving safety.^5^ Individualised AUC_24_/MIC targeting is not readily available in LMICs but has been successfully used as an entry point for stewardship elsewhere.^14^

Paediatric prescribing practices of vancomycin have not been evaluated in South Africa. To identify potential AMS intervention opportunities related to vancomycin usage and to aid better patient outcomes, the authors aimed to document the use, prescribing practices and monitoring of intravenous vancomycin, and to describe the spectrum of bacteria isolated on microbiological culture and their antibiotic susceptibility profile in children treated with intravenous vancomycin during a 12-month period at Red Cross War Memorial Children’s Hospital (RCWMCH) in Cape Town, South Africa.

## Methods

### Study design, setting and identification of study participants

A retrospective descriptive study of intravenous vancomycin use in children admitted to RCWMCH during 2019 was performed. The RCWMCH is a 272-bed referral hospital that serves children aged 13 years and younger from the Western Cape province and occasionally from surrounding provinces. All children treated with intravenous vancomycin during the study period were eligible for inclusion and excluded only if records were missing or the prescription chart could not be located in the hospital folder. Study participants were primarily identified from the RCWMCH pharmacy database. In addition, the intensive care unit (ICU) discharge summary database was searched, as vancomycin used to treat children admitted to the ICU is obtained from ICU medication stock and not issued directly from the pharmacy on an individual patient basis.

### Data collection

Demographic details, clinical information and vancomycin prescription data on the enrolled participants were extracted from medical records obtained from the RCWMCH medical records department and entered into a REDCap database (Research Electronic Data Capture, Vanderbilt University, Nashville, Tennessee, United States [US]). The collected clinical information included diagnosis, comorbidities, surgical interventions, documentation of adverse drug events (ADEs) and clinical outcomes. Laboratory information collected included vancomycin trough concentrations and creatinine at the time of vancomycin administration. Details of each vancomycin prescription episode were obtained from the paper-based antibiotic prescription chart (a separate prescription chart from that used for other medication) that is currently in use at RCWMCH. This chart prompts the prescriber to send appropriate cultures prior to antibiotic prescribing and to indicate the suspected clinical diagnosis, source of infection, and an indication as to whether the antibiotic is for empirical or directed treatment. The route, dose, dosing frequency, duration, and start date and time are also captured (Online Appendix 1). Prescription data were collected directly from this antibiotic chart where available, and multiple prescription episodes were collected within a single admission. The total patient days for all hospital admissions during the study period was calculated by and received directly from the Provincial Health Data Centre.

The collected microbiological information included culture site, organism(s) cultured, antibiotic susceptibility testing (AST) results, and the MIC to vancomycin. All microbiological cultures were performed at the National Health Laboratory Service (NHLS) microbiology laboratory at Groote Schuur Hospital in Cape Town. Bacterial identification and AST were performed using the Vitek^®^2 automated system (bioMérieux, France), biochemical or antigen-detection methods, and disc or gradient diffusion AST methods where appropriate. Results of AST were interpreted using the Clinical & Laboratory Standards Institute (CLSI) guidelines.^15^

### Data analysis

Data were analysed using Microsoft Excel (version 2111) and Stata version 15.0 (Stata Corp., College Station, Texas, US). Categorical variables were described using absolute values and percentages with the associated 95% confidence intervals (CIs); continuous variables were described using median and interquartile range (IQR) for non-normally distributed data. Comparison of the vancomycin daily dose between treatment groups was done using the Wilcoxon rank sum test. To examine whether participants with a Gram-positive organism on culture had increased odds of being prescribed vancomycin for more than 14 days compared to those with negative cultures, odds ratio (OR) were calculated using the STATA immediate command. *P*-values of less than 0.05 were used to denote statistical significance.

### Study definitions

An infection episode was considered community-acquired (CAI) if vancomycin was started within 48 h of admission to hospital and considered hospital-acquired (HAI) if vancomycin treatment was started more than 48 h after hospital admission or within 30 days of discharge from a previous admission.^16^

A vancomycin prescription was considered empiric treatment if commenced before culture and antibiotic susceptibility results were available, and all prescriptions for which cultures remained negative or no cultures were submitted to the laboratory. A vancomycin prescription was considered directed when an organism susceptible to vancomycin was identified by culture and AST, and vancomycin was considered the preferred treatment option.

Vancomycin usage was expressed as days of therapy per 1000 patient days (DOT/1000 PD) defined as the number of days that study participants were treated with intravenous vancomycin (regardless of the dose) per 1000 inpatient days including all patients admitted in the hospital during the study period.^17^

Vancomycin TDM was considered appropriate if a serum trough concentration was performed before the fourth dose for treatment exceeding 3 days, based on the time to reach steady state.^18^ Trough concentrations of 10 mg/L – 20 mg/L were considered therapeutic based on guidelines followed within the study period.^16,18^

### Ethical considerations

Ethical approval was obtained from the University of Cape Town Human Research Ethics Committee (approval number HREC Ref: 498/2020) and the RCWMCH research committee (RCC 241/WC_202008_114). A waiver of consent was granted on the basis that the study only used anonymised archived data. The study was done in accordance with the Declaration of Helsinki.

## Results

**FIGURE 1:**
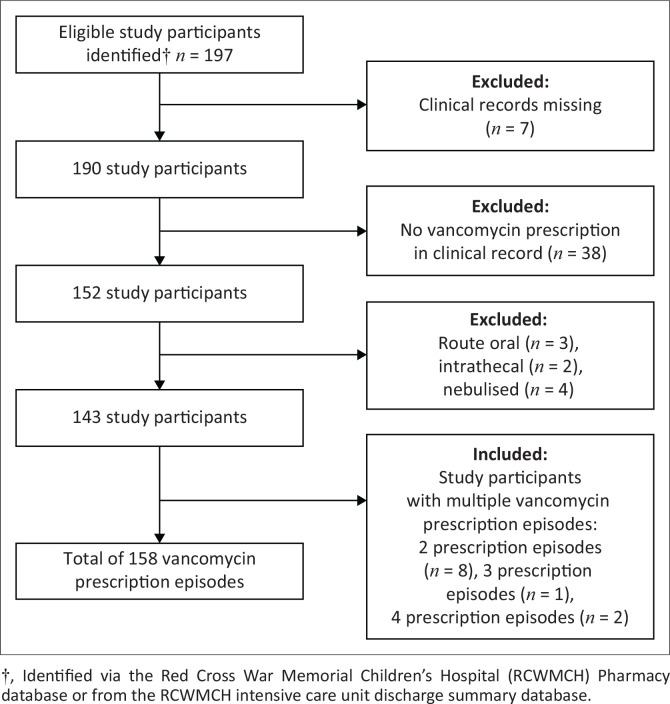
Participants and vancomycin prescription episodes included in the study.

**FIGURE 2:**
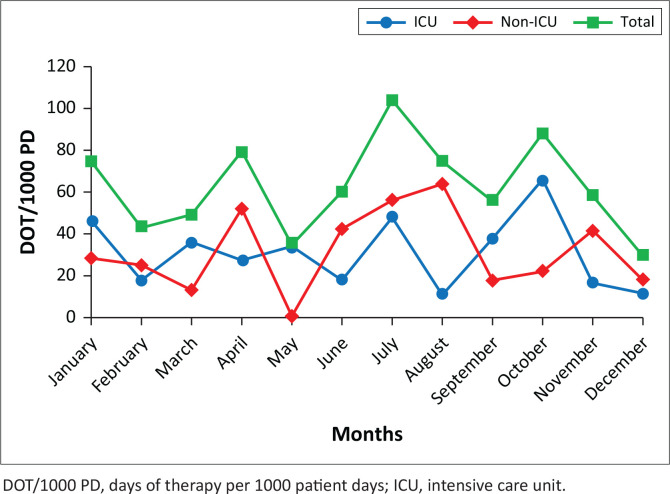
Monthly usage of vancomycin over the study period (2019) in ICU & non-ICU wards at the Red Cross War Memorial Children’s Hospital.

A total of 158 vancomycin prescription episodes for 143 children were included (Figure 1), of which 67 (47%) were male and 76 (53%) were female. The median age at admission was 7 months (IQR: 1–77) and the median weight was 7 kg (IQR: 3–19). Twenty-two (15%) of participants were term neonates and 26/143 (18%) had been born premature with a corrected gestational age below 44 weeks. Monthly usage stratified by use in and outside of the ICU is illustrated in Figure 2.

### Vancomycin usage

The overall usage of intravenous vancomycin during the study period was 63 DOT/1000 PD (IQR: 38–72). The monthly usage ranged from 1 DOT/1000 PD to 104 DOT/1000 PD. Vancomycin prescription episodes occurred in the following wards: ICU (81/158, 51%), transplant (23/158, 15%), surgical (19/158, 12%), haematology/oncology (19/158, 12%) and general paediatric (16/158, 10%).

### Vancomycin prescribing practices

#### Diagnosis and indications for vancomycin

All vancomycin prescription episodes met the definition of an HAI. Most prescriptions (127/158, 80%) occurred more than 48 h after admission. For the 31 episodes in which vancomycin treatment was started before 48 h, 19 (61%) were transferred in from other hospitals where they had more than 48 h, and 12 (39%) were discharged within 30 days prior to current admission. Vancomycin was prescribed as empiric treatment in 127/158 (80%) prescription episodes and directed treatment for 31/158 (20%). Suspected clinical diagnoses and indications for vancomycin use are summarised in Table 1.

**TABLE 1:** Clinical classification associated with each vancomycin prescription episode.

Clinical classification†	Empiric (*n* = 127‡)			Directed (*n* = 31‡)		
	*n*	%	95% CI	*n*	%	95% CI
**Infection without a clinical focus**						
Fever§	4	3	1–8	1	3	0–17
Neutropaenia¶	7	6	2–11	2	6	1–21
**Infection with a clinical focus**						
Hospital acquired pneumonia	13	10	6–17	2	6	1–21
Ventilator associated pneumonia	18	14	9–21	4	13	4–30
Shunt infection††	3	2	0–7	-	-	-
Subdural empyema	1	1	0–4	-	-	-
Ventriculitis	3	2	0–7	-	-	-
Meningitis	6	5	2–10	-	-	-
Intra-occular infection	-	-	-	1	3	0–17
Intra-abdominal infection‡‡	20	15	10–23	4	13	4–30
Necrotizing enterocolitis	14	11	6–18	3	10	2–26
Typhlitis	2	2	0–6	-		-
Skin and soft tissue infection§§	24	19	12–27	2	6	1–21
Toxic shock syndrome	1	1	0–4	-	-	-
Osteitis	-	-	-	1	3	0–17
Spinal Abscess	1	1	0–4	-	-	-
Urinary tract infection	3	2	0–7	3	10	2–26
Infective endocarditis	2	2	0–6	-	-	-
Central line infection	5	4	1–9	7	24	10–41
Blood stream infection	-	-	-	1	3	0–17

† , Includes any clinical diagnosis supplied at vancomycin initiation;

‡ , *n* = denominator unless otherwise specified;

§ , Body temperature > 38 ⁰C at the time of prescription;

¶ , Absolute neutrophil count < 1.5 X 109/L;

†† , Includes ventriculoperitoneal and Ommaya shunts;

‡‡ , Includes all post-surgical intra-abdominal infections and peritonitis;

§§ , Includes skin abscesses, burn wound infections and wound sepsis.

#### Dose

A starting dose of 15 mg/kg per dose was identified in 83/158 (53%) prescription episodes, with the median starting dose also being 15 mg/kg per day (IQR: 14–15). The median daily dose was 45 mg/kg per day (IQR: 43–60) with no significant difference between the median total daily dose in the definitive 46 mg/kg per day (IQR: 40–60) versus empiric treatment 45 mg/kg per day (IQR: 43–60) groups (*z* = 0.3, *p* = 0.8). Thirty-four (22%) prescriptions were for a total daily dose of 60 mg/kg per day, and 46/158 (29.1%) of the prescriptions were for a total daily dose of ≥ 60 mg/kg per day.

#### Dosing frequency

Dosing frequencies included six-hourly dosing in 42/158 (27%) and eight-hourly dosing in 89/158 (56%) prescription episodes. Other frequencies included 12-hourly dosing in 11/158 (7%) of which 10 (10/11, 90%) frequencies were based on prematurity and gestational age, and 1 (1/11, 10%) had renal dysfunction; 3 (3/158, 2%) episodes of 24-hourly dosing in which 1 (1/3, 33%) was based on gestation, and 2 (2/3, 67%) had renal dysfunction. In both episodes (2/158, 1%) where vancomycin was prescribed 48-hourly, renal dysfunction was present. A single dose was given in 11/158 (7%) episodes with 10 (10/11, 90%) stopped after a single dose and 1 (1/11, 10%) episode where the vancomycin was represcribed eight-hourly.

#### Duration of treatment

The median duration of vancomycin treatment was 5 days (IQR: 3–7). There was a significant difference in treatment duration between directed (7 days; IQR: 4–10) and empiric (4 days; IQR: 2–7) vancomycin treatment groups (*z* = 3.2, *p* = 0.001). In 15/158 (9%) prescription episodes, the duration of vancomycin treatment was 14 days or more. These prescriptions included empiric treatment of suspected meningitis or ventriculitis (4/15, 26%), skin and soft tissue infections (SSTIs) (2/15, 13%), postsurgical intra-abdominal infection and typhlitis (2/15, 13%), ventilator-associated pneumonia (VAP) (1/15, 7%), febrile neutropaenia (1/15, 7%), spinal abscess (1/15, 7%) and suspected toxic shock syndrome (1/15, 7%). One episode of necrotising enterocolitis (NEC) and coagulase-negative staphylococci (CONS) on blood culture (1/15, 7%) and two episodes of directed treatment for confirmed MRSA BSI (2/15, 13%) from wound sepsis after cardiac surgery and hospital-acquired pneumonia (HAP) in an episode of flame burns. In 3 of 15 episodes (20%) where vancomycin was prescribed for 14 days or more, Gram-positive organisms were cultured. Similarly, in episodes where vancomycin was prescribed for less than 14 days, 28/143 (20%) isolated Gram-positive bacteria from culture. Thus, the odds of having a Gram-positive organism on culture when treated with vancomycin for 14 days or more compared to the odds when treated for less than 14 days were similar (OR: 1.02, 95% CI: 0.17–4.2, *p* = 0.59).

### Therapeutic drug monitoring

Therapeutic drug monitoring was performed in 95/158 (60%) of the vancomycin prescription episodes. Vancomycin treatment exceeded 3 days in 98/158 (62%) prescription episodes, and of these, 65/98 (66%) had vancomycin serum trough concentrations performed. The median time of trough concentration collection was before the fourth dose (IQR: 3–5).

### Dosing strategies in relation to vancomycin serum trough concentrations

For samples obtained before the fourth dose, the median vancomycin serum trough concentration was 14 mg/L (IQR: 6–18).

There were 37/95 (39%) serum trough concentrations < 10 mg/L. Irrespective of the timing of serum trough concentration measurements, prescription adjustments based on trough concentrations below 10 mg/L included an increase in dose (20/37, 54%), dose frequency (13/37, 23%) or both (5/37, 14%). Nine (9/37, 24%) prescriptions were not adjusted.

Trough concentrations greater than 20 mg/L occurred in 21/95 (22%) of all prescription episodes where a trough concentration was performed. The dose (6/21, 29%), frequency (9/21, 43%) or both (1/21, 4%) were adjusted. Vancomycin was stopped in 3 (3/21, 14%) and continued without any adjustments in 2 (2/21, 10%) episodes.

### Microbiology

Cultures were done before initiating 146/158 (92%) vancomycin prescription episodes. Culture results are detailed in Table 2. A high proportion of culture-positive episodes had an indwelling device present (39/58, 67%). These included indwelling venous catheters (31/58, 53%), urinary catheters (4/58, 7%), intracranial monitors (2/58, 3%), a ventriculoperitoneal shunt (VPS) (1/58, 2%) and a peritoneal dialysis catheter (1/58, 2%). Of the 17 *S. aureus* isolates, 8 (47%) were MRSA. There were 15 *Enterococcus* spp. isolated, 13 of which (87%) were resistant to ampicillin. Vancomycin MICs were performed on 21/38 (55%) of the Gram-positive isolates including all 8 MRSA isolates. All MICs were ≤ 1 mg/L.

**TABLE 2:** Microbiological profile of organisms isolated from positive cultures.

Sample type (*n* = 58‡)	*n*	%	Organisms identified (*n* = 61‡)	*n*	%
Blood	46†	79	*Staphylococcus aureus*	13	21
	-	-	Coagulase negative *Staphylococcus*	8	13
	-	-	*Enterococcus faecalis*	2	3
	-	-	*Enterococcus faecium*	10	16
	-	-	*Streptococcus mitis*	2	3
	-	-	*Streptococcus viridans*	2	3
	-	-	*Streptococcus agalactiae* §	1	2
	-	-	Gram-negatives¶	6	10
	-	-	Candida species††	4	7
Pus swabs	4	8	*Staphylococcus aureus*	2	3
	-	-	*Enterococcus faecium*	1	2
	-	-	*Klebsiella pneumoniae*	1	2
Respiratory samples	3	5	*Staphylococcus aureus*	2	3
	-	-	*Serratia marcescens*	1	2
Peritoneal fluid	2	3	Coagulase negative *Staphylococcus*	1	2
	-	-	*Enterococcus faecium*	1	2
Urine	2	3	*Enterococcus faecium*	1	2
	-	-	*Enterococcus species* ‡‡	1	2
Vitreous fluid	1	2§§	*Streptococcus salivarius*	1	2
	-	-	*Streptococcus mitis*	1	2

† , Total pathogens isolated from blood culture includes two isolates identified in two cultures (48 pathogens from 46 samples), including a mixed growth of *Klebsiella pneumoniae* and *Escherichia coli* and *Staphylococcus aureus* and *Streptococcus mitis*;

‡ , n = denominator unless otherwise stated;

§ , Group B Streptococcus;

¶ , *Klebsiella pneumoniae, Citrobacter freundii, Serratia marcescens, Pseudomonas aeruginosa, Klebsiella oxytoca, Escherichia coli*;

†† , *Candida albicans, Candida parapsilosis*;

‡‡ , Only identified up to genus;

§§ , Two pathogens isolated from one vitreous fluid sample.

### Antibiotic de-escalation practices following culture and antibiotic susceptibility results

Cultures were negative in 88/146 (60%) episodes and the median vancomycin treatment duration in these episodes was 5 (IQR: 3–6) days. Vancomycin was stopped within 3 days for 36/88 (41%), within 5 days for 28/88 (32%) and continued for a median of 7 (IQR: 6–10) days for 24/88 (27%) prescription episodes.

Positive cultures were obtained in 58/146 (40%) vancomycin prescription episodes and the median vancomycin treatment duration in these episodes was 6 (IQR: 2–8) days. Vancomycin was the appropriate antibiotic choice and prescribed as directed treatment in 22/58 (38%) prescription episodes and 9/58 (15%) episodes where CONS were identified and of which the clinical significance was uncertain. For 19/58 (33%) episodes, vancomycin was stopped within 5 days as culture either identified an organism for which vancomycin was not the appropriate antibiotic choice or the organism was not deemed clinically significant.

Vancomycin was continued beyond 5 days in 8/58 (14%) when the organism identified was either not susceptible to vancomycin or vancomycin was not considered the preferred treatment option. These included the following: 3/8 (37%) episodes where Gram-negative bacteria were isolated from blood and pus in suspected NEC^2^ and from a tracheal aspirate in febrile neutropaenia^1^; 3/8 (37%) episodes where methicillin-susceptible *S. aureus* was identified from sputum in an episode of VAP, from a pus swab from a wound infection following cardiac surgery, and from blood culture in febrile neutropaenia, respectively; 1 (1/8, 13%) episode in which *Candida albicans* was isolated from blood culture in an episode of VAP; and 1 (1/8, 13%) episode in which *Streptococcus agalactiae* was isolated from blood culture in an episode of NEC.

### Adverse events

Four ADEs were documented in the clinical records. One episode was associated with a hypersensitivity reaction; three had acute kidney injury (AKI) attributed to vancomycin with all having preexisting chronic kidney disease (advanced membranous nephropathy, lupus nephritis and hepatorenal syndrome). Two participants with documented AKI also received furosemide and amikacin, respectively. Whether co-administration of these drugs contributed to AKI is uncertain. Vancomycin TDM and dose adjustment were performed throughout the course of therapy for participants with ADE. Vancomycin was stopped in two (50%) episodes and continued despite the ADE in two (50%) episodes.

## Discussion

To the best of the authors knowledge, this is the first description of vancomycin usage and prescribing practices in South African children. Intravenous vancomycin prescription occurs throughout RCWMCH, with ICU unsurprisingly accounting for most usage (51%) during 2019. Comparable vancomycin usage has not been published from healthcare facilities treating children in South Africa.

Vancomycin was prescribed exclusively for HAI and mostly as empiric treatment (80%) and less often as directed treatment (20%) with cultures routinely obtained prior to prescriptions in most (92%). Clinical indications for using vancomycin at initiation of empiric antibiotic treatment included intra-abdominal infection and NEC (26%), HAP and VAP (24%) and SSTI (19%). For directed treatment, indwelling venous catheter-associated infection (24%), HAP and VAP (19%), and intra-abdominal infection and NEC (23%) were common. Vancomycin prescription for inappropriate or non-approved indications was less frequent than previously described in other settings.^7^

Dosing of vancomycin was variable with only 53% of prescriptions adhering to a starting dose of 15 mg/kg per day and only 22% adhering to the recommended total daily dose of 60 mg/kg per day.^19^ More than two-thirds of prescriptions were for < 60 mg/kg per day. Dosing frequency was most often eight-hourly (56%) or six-hourly (27%) with dosing less frequently than eight-hourly due to prematurity or renal dysfunction.

The significant difference in treatment duration between empiric and directed treatment episodes suggests that vancomycin is stopped sooner once cultures are negative than in cases where cultures and AST confirm an infection where vancomycin is the appropriate treatment. However, among the 15 episodes (9%) where vancomycin treatment continued for 14 days or more, empiric treatment accounted for 80% of prescription episodes. These findings are in keeping with studies from other countries indicating that inappropriate prescribing and prolonged empiric treatment are common, with differences among age groups contributing to dosing complexity.^20,21,22^

The optimal dosing of vancomycin and the role of TDM in paediatric practice remain uncertain^11,12^ and may soon be replaced with model-informed precision dosing where available.^23^ Infectious Diseases Society of America guidelines^5^ recommend AUC_24_/MIC targeting, whereas guidelines aiming for vancomycin serum trough concentrations of 10 mg/L – 20 mg/L are still followed at RCWMCH.^19^ Serum trough concentration monitoring was only performed in 60% of prescription episodes overall and in 66% of episodes treatment for longer than 3 days. Only 21% of TDM samples were obtained before the fourth dose, lower than the 40% reported in a study from France that evaluated 154 vancomycin prescriptions.^24^ With the very variable dosing found in the study overall, it is surprising that the median vancomycin serum trough concentration in the 20 episodes with samples obtained before the fourth dose was 14 mg/L (recommended range 10 mg/L – 20 mg/L)^19^ although the variability was wide (IQR: 6–18). It was difficult to interpret the serum trough concentrations that were < 10 mg/L or > 20 mg/L, as well as the appropriateness of dose adjustments done in response to these results. Although AUC_24_/MIC monitoring is not currently available, its future utilisation as an AMS tool may improve vancomycin dosing and safety, which is particularly important as ADEs are likely underreported at RCWMCH.^14^

The microbiological results revealed that approximately two-thirds of positive cultures occurred where an indwelling device was present. This finding suggests that improved compliance with infection prevention and control (IPC) could reduce the need for vancomycin prescription. Although blood culture yielded MRSA in 47% of isolates, it is reassuring that no isolates were non-susceptible to vancomycin. The rate of MRSA in was lower than the 72% and 65% in hospital-acquired BSI due to *S. aureus* recorded in previous paediatric studies done in Cape Town.^25,26^

Like many others in LMIC settings, RCWMCH lacks the capacity to implement prospective audit and real-time feedback to prescribers, and discontinuation or de-escalation to narrower spectrum antibiotics based on culture and AST results where appropriate. Among episodes with negative cultures, vancomycin was discontinued in 41% within 3 days and 73% within 5 days of starting treatment. However, among 58 episodes with positive cultures, 8 (14%) continued vancomycin treatment despite the identification of an organism that was not susceptible to vancomycin or where vancomycin is not the preferred treatment option.

This study has several limitations. It was performed at a single centre and paper-based medical record review with missing documents resulted in a high exclusion rate (23%). Uniform local guidance on vancomycin prescribing is limited and variable, and the appropriateness of vancomycin treatment based on the documented clinical syndrome could not be assessed. Clinical indications were only collected when vancomycin was initiated, as information on indications for continued prescribing was not available. Furthermore, outcome data were not available and the impact of vancomycin prescription errors on outcomes could not be accurately assessed. As a result of the diversity in prescribing practices among different institutions, results may not be applicable elsewhere.

Monitoring antimicrobial consumption and instituting effective AMS interventions remain a challenge in areas where electronic prescribing is not yet feasible. This study emphasises the need to develop and implement uniform guidelines for the prescription and monitoring of vancomycin along with appropriate de-escalation practices as urgent AMS interventions. Similar guidance is important for all other antimicrobials prescribed at RCWMCH. Ongoing education of prescribers and compliance with IPC practices are essential. Future research should focus on defining methods to improve prescribing practices and evaluate future AMS interventions.

## Conclusion

Dosing errors, prolonged empiric treatment and inappropriate vancomycin monitoring were problems associated with vancomycin prescriptions in the study. Antimicrobial stewardship interventions targeting uniform guidance and concurrently improving IPC compliance may contribute to the preservation of vancomycin at RCWMCH.
